# An active IGF-1R-AKT signaling imparts functional heterogeneity in ovarian CSC population

**DOI:** 10.1038/srep36612

**Published:** 2016-11-07

**Authors:** Ram K. Singh, Ajit Dhadve, Asmita Sakpal, Abhijit De, Pritha Ray

**Affiliations:** 1 Imaging Cell Signaling and Therapeutics Lab, Advanced Centre for Treatment, Research and Education in Cancer (ACTREC), Tata Memorial Centre, Navi Mumbai, Maharashtra, India; 2 Molecular Functional Imaging Laboratory, Advanced Centre for Treatment, Research and Education in Cancer (ACTREC), Tata Memorial Centre, Navi Mumbai, Maharashtra, India

## Abstract

Deregulated IGF-1R-AKT signaling influences multiple nodes of cancer cell physiology and assists in migration, metastasis and acquirement of radio/chemoresistance. Enrichment of cancer stem cells (CSC) positively correlates with radio/chemoresistance development in various malignancies. It is unclear though, how IGF-1R-AKT signalling shapes CSC functionality especially in ovarian cancer. Previously we showed that upregulated IGF-1R expression is essential to initiate platinum-taxol resistance at early stage which declines with elevated levels of activated AKT at late resistant stage in ovarian cancer cells. Here, we investigated the effect of this oscillatory IGF-1R-AKT signalling upon CSC functionality during generation of chemoresistance. While gradual increase in CSC properties from early (ER) to late (LR) resistant stages was observed in three different (cisplatin/paclitaxel/cisplatin-paclitaxel) cellular models created in two ovarian cancer cell lines, the stemness gene expressions (*oct4*/*sox2*/*nanog*) reached a plateau at early resistant stages. Inhibition of IGF-1R only at ER and AKT inhibition only at LR stages significantly abrogated the CSC phenotype. Interestingly, real time bioluminescence imaging showed CSCs of ER stages possessed faster tumorigenic potential than CSCs belonging to LR stages. Together, our data suggest that IGF-1R-AKT signalling imparts functional heterogeneity in CSCs during acquirement of chemoresistance in ovarian carcinoma.

Insulin like Growth Factor-1 Receptor (IGF-1R) is a transmembrane receptor tyrosine kinase which transmits signal via PI3K-AKT or MAPK-ERK pathways^1^,^2^,^3^. In addition to its essential functions for normal growth and development, deregulated IGF-1R signaling plays a major role in tumor growth and chemoresistance^4^,^5^,^6^. Generation of radio/chemoresistance is a major hurdle in successful treatment of cancers which may arise due to presence of inherently resistant tumor cells or due to acquirement of resistance by these cells^5^,^7^,^8^. While molecular alteration in various pathways assist in resistance development^9^, a small subset of inherently resistant cells within tumor bulk known as Cancer Stem Cells (CSC) also aid in acquirement of chemoresistance and relapse^10^,^11^,^12^. Currently considerable effort is undergoing to develop strategies to target these deadly populations for ultimate cure of cancer. Historically CSCs from different malignancies are isolated using a set of biomarkers. However, overlapping presence of these biomarkers in normal cell types poses a real challenge for targeting CSCs. In congruence with intratumoral heterogeneity, recent evidences suggest that CSCs are also not uniform but rather heterogeneous population and highly plastic in nature^13^,^14^,^15^. Existence of such heterogeneity within CSCs adds another layer of complexity for efficient targeting. Till date, CSC heterogeneity has been recognized by presence of biomarkers along with certain functional assays. CD44+/CD24^−^ and ALDH + breast CSCs are reported to be more tumorigenic with poor clinical outcome than CSCs expressing CD44+/CD24^−^ alone^16^. Additionally, both CD133^+^ and CD133^−^ CSC population from glioblastoma tumors found to possess self-renewing and tumor-initiating properties thereby casting doubt on biomarker based CSC isolation and characterization^17^. Still biomarker based therapeutic strategy to eradicate CSCs was attempted and patient derived CD44 + ovarian CSCs possessing high claudin-4 expression were shown to be effectively targeted by *Clostridium perfringens* enterotoxin^18^. Intriguingly, whether and how a signaling pathway bestows heterogeneity in CSC population has so far not been investigated.

Using indigenously developed resistant models against cisplatin, paclitaxel and dual drugs in ovarian cancer cells, we showed that upregulated IGF-1R expression is crucial to initiate resistance and an activated AKT later assists in maintenance of resistance^19^,^20^. Irrespective of nature of drugs, early resistant (ER) cells of all these models show higher IGF-1R expression, while late resistant (LR) cells possess low IGF-1R but elevated phosphorylated AKT^19^. Role of IGF-1R in developing cisplatin or paclitaxel resistance in ovarian cancer cells were reported by others^5^,^6^.

Herein we investigated the consequence of this oscillatory IGF-1R-AKT signaling upon CSC properties during acquirement of platinum-taxol resistance. While gradual increase in CSC features were found to be positively correlated with resistance development (from ER to LR stages), the stemness gene expressions reached a plateau early on. Inhibition of IGF-1R at ER and AKT inhibition at LR stages significantly abrogated CSC and chemoresistant phenotype. Interestingly, real time imaging showed CSCs of ER stages possessed higher and faster tumorigenic potential than CSCs belong to LR stages. Inhibition of AKT relieved IGF-1R suppression and sensitized the late resistant cells to combinatorial treatments. This is the first report on an intricate and interdependent relation between IGF-1R and AKT with functional heterogeneity of ovarian cancer stem cells which might emerge as a therapeutic target for the resistant disease.

## Results

### Enrichment of Stem cell like features with acquirement of drug resistance in ovarian cancer cells

We have previously developed dynamic models of drug resistance against cisplatin, paclitaxel and both drugs by treating A2780 and OAW42 ovarian cancer cell lines with successive and gradually incrementing drug concentration and categorized them into early (ER) (Cis^ER^, Pac^ER^ and Dual^ER^)and late (LR) (Cis^LR^, Pac^LR^ and Dual^LR^) resistant stages depending on their resistant indices^20^. Intriguingly, irrespective of the nature of drugs, elevated levels of IGF-1R and high phosphorylated AKT were found to be associated with early and late stages of resistance which seem to be essential for initiation (at early stage) and maintenance (late stage) of drug resistance^19^.

**Figure 1:**
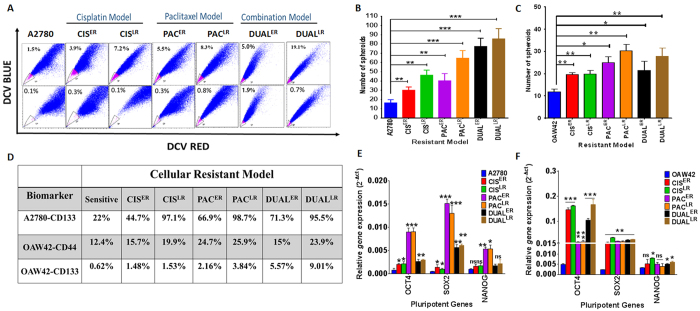
Characterization of stem cell like features across the cellular resistant models. (**A**) Increased Side Population in three different A2780 resistant models (cisplatin, paclitaxel and dual). FACS dot plot showing the distribution of SP cells with or without verapamil treatment which increases with increasing resistance. (**B**,**C**) Graphical representation of enhanced spheroid formation observed across all the resistant models. Resistant models in OAW42 cells exhibited slightly lesser spheroids (Cis-Res = 1.63–1.66 fold; Pac-Res = 2.08–2.5 fold; Dual-Res = 1.8–2.3 fold) but resistant models of A2780 cells showed considerably elevated spheroid formation (Cis-Resistant model = 1.8–2.8 fold; Pac-Resistant model = 2.4–3.9 fold; Dual-Resistant model = 3.8–5.3 fold) than the respective sensitive cells. (**D**) Increased expression of biomarkers (CD133 and CD44) with enhanced resistance observed across the resistant models. Control A2780 cells showed 22% positivity for CD133 expression which increased in each of the drug resistance models; (CD133expression: A2780 = 22%; Cis^ER^ = 44.7%; Cis^LR^ = 97.1%; Pac^ER^ = 66.9%; Pac^LR^ = 98.7%; Dual^ER^ = 71.3%; Dual^LR^ = 95.5%). A2780 cells did not show detectable CD44. CD44 expression in OAW42 cellular models also showed increments with increasing resistance (OAW42 = 12.7%; Cis^ER^ = 15.7%; Cis^LR^ = 19.9%; Pac^ER^ = 24.7%; Pac^LR^ = 25.9%; Dual^ER^ = 15%; Dual^LR^ = 23.9%). Low but increasing CD133 expression was associated with OAW42 resistant models (OAW42 = 0.62%; Cis^ER^ = 1.48%; Cis^LR^ = 1.53%; Pac^ER^ = 2.16%; Pac^LR^ = 3.84%; Dual^ER^ = 5.57%; Dual^LR^ = 9.01%) (**E,F**) Real time quantification of *oct4, sox2* and *nanog* expression across A2780 resistant models showed maximal expression at early resistant stages which remained constant till late resistant stages. Similar trend was observed in OAW42 resistant models.

To understand the association of Cancer Stem Cell dynamics with acquirement of resistance, functional assays and biomarker association were studied in these cellular resistant models. Side population assay (SP) which purifies CSCs based on their innate drug efflux property was used for CSC isolation from different stages of resistance. A gradual and significant enrichment in SP cells (3.9 ± 0.05% in Cis^ER^ & 7.2 ± 0.42% in Cis^LR^ cells) compared to the chemosensitive A2780 cells (1.5 ± 0.05% SP) was observed in cisplatin resistant model (p < 0.05). Similar enhancement in SP cells was observed with both Paclitaxel and dual resistant models. The dual resistant model showed maximal enrichment in SP population at late resistant stage (19.1 ± 1.0% (13.2 fold) (Fig. 1A). The OAW42 resistant models also exhibited enhanced SP population across cisplatin, paclitaxel and dual resistant cells compared to OAW42 sensitive cells (Supplementary Table 2). However, the absolute level of SP cells was 2-fold higher in A2780-Dual^LR^ than OAW42-Dual^LR^ cells (19.1% vs. 9.8%). Cancer stem cells possess higher self-renewal ability which is assessed through spheroid formation assay. In both A2780 and OAW42 cellular resistant models, significant enhancement in spheroid formation was observed with increasing resistance. When compared between the models, both Pac^ER^ and Pac^LR^ cells exhibited enhanced spheroid forming ability than Cis^ER^ and Cis^LR^ cells. However, this trend only met significance for Pac^LR^ cells (Fig. 1B,C). It was also observed that both sensitive cells (A2780 and OAW42) cells could form spheroids up to two passage only, while resistant cells were capable of forming spheroids till seven passages. Higher spheroid formation in A2780 resistant models suggests superior self-renewal ability of these cells compared to OAW42 cells.

The level of known ovarian CSC biomarkers (CD44 and CD133) expression was monitored in both A2780 and OAW42 resistant models (Fig. 1D, Supplementary Figure 1). A2780 cells did not show detectable CD44 expression (data not shown) however the level of CD133 showed marked increase with increasing resistance (A2780 = 22%; Cis^ER^ = 44.7%; Cis^LR^ = 97.1%; Pac^ER^ = 66.9%; Pac^LR^ = 98.7%; Dual^ER^ = 71.3%; Dual^LR^ = 95.5%). We also tested the expression of these markers in OAW42 cellular resistant models where CD44 expression showed marked increase with increasing resistance (OAW42 = 12.7%; Cis^ER^ = 15.7%; Cis^LR^ = 19.9%; Pac^ER^ = 24.7%; Pac^LR^ = 25.9%; Dual^ER^ = 15%; Dual^LR^ = 23.9%). Very low expression of CD133 was observed in OAW42 cisplatin and paclitaxel resistant models compared to A2780 resistant models which showed the similar trend of enhanced expression with increasing resistance. The dual resistant model in OAW42 cells showed maximum enhancement in CD133 level (OAW42 = 0.62%; Cis^ER^ = 1.48%; Cis^LR^ = 1.53%; Pac^ER^ = 2.16%; Pac^LR^ = 3.84%; Dual^ER^ = 5.57%; Dual^LR^ = 9.01%) (Fig. 1D, Supplementary Figure 1). Interestingly, real time quantification of *oct4, sox2* and *nanog* (Pluripotent genes) expression showed marked increase at early resistance stages compared to the sensitive stages which remained unaltered even at late resistant stages uniformly in all the resistant models (Fig. 1E,F).

### Side population fraction is enriched with cancer stem cell features

**Figure 2:**
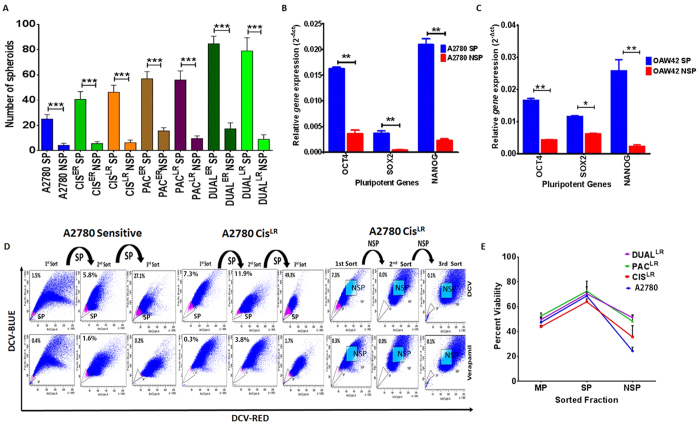
Characterization of side population cells for their stem cell like features. (**A**) Graphical representation of higher spheroid forming ability of SP cells of A2780 sensitive, early and late resistant cells from all the three resistant models than corresponding NSP cells. (**B**,**C**) Real time quantification of *oct4, sox2* and *nanog* expression in SP and NSP fractions of A2780 and OAW42 sensitive cells showing significantly increased expression of these pluripotent genes in SP cells than NSP cells. (**D**) FACS analysis of differentiating ability of SP and NSP cells monitored over three subsequent passages by DCV staining. (**E**) MTT assay showing marked increase in viability of SP cells (A2780 SP = 68.75%, Cis^LR^ SP = 63.99%, Pac^LR^ SP = 72.4% and Dual^LR^ SP = 70.45%) compared to main population (MP) and NSP cells at IC_50_ of respective drug concentration. (Data represented as ± SEM; *p < 0.05; **p < 0.005; ***p < 0.0005).

Side population (SP) assay is considered a routine method for CSC characterization. However, the discriminating power of this assay to identify stem cells over non-stem cell population is sometimes criticized^21^. To test the true stemness phenotype of the SP population of all the resistant models (A2780 & OAW42), both self-renewal and differentiation properties were assayed. SP cells sorted from sensitive and early and late resistant stages of all three drug resistant models of A2780 showed significantly higher spheroid formation (p < 0.05) than their non-SP counterparts (Fig. 2A). Interestingly, not much difference in spheroid forming ability was found between the early and late resistant cells of each drug resistant models (Fig. 1A,B). When compared across the models, an apparent enhanced spheroid formation was observed for Paclitaxel resistant cells than Cisplatin resistant cells. However, only Pac^LR^ cells formed significantly higher number of spheroids compared to Cis^LR^ cells (p < 0.05). The spheroid forming ability between Cis^ER^ and Pac^ER^ cells did not show any significant difference.

Transcript levels of *oct4, sox2* and *nanog* were significantly higher in SP cells than their NSP counterparts (Fig. 2B,C). *In vitro* differentiation is one of the important characteristic features to asses CSC phenotype. We performed serial sorting of SP and NSP cells from chemosensitive and late cisplatin-resistant A2780 cells for three cycles. Intriguingly, we did not observe an absolute persistence of 100% SP cells from 1^st^ to 3^rd^ sort, rather a gradual enrichment (A2780~1.4% to 27.1%; A2780 Cis^LR^~7.3% to 49.3%) was found and simultaneously the NSP population decreased. The NSP cells, in contrary did not form any SP cells till third passage (Fig. 2D). In addition to stemness phenotype we also measured the resistant phenotype where SP cells of late resistant models of A2780 showed higher viability at respective IC_50_ concentrations (CIS^LR^ = 5 ug/ml; PAC^LR^ = 125 ng/ml and DUAL^LR^ = 500 ng/ml cisplatin+ 80 ng/ml paclitaxel) compared to their NSP or main population. The slight difference in survival rate between MP and NSP population suggests that NSP cells can maintain their chemoresistance phenotype but are devoid of stem cell pool (Fig. 2E). We also measured cellular viability of SP, NSP and MP fractions for every cycle during enrichment of SP cells and found that SP cells showed significantly higher viability compared to its respective counterparts (MP and NSP cells) (Supplementary Figure 3).

### Real time monitoring of tumor growth kinetics of SP population by bioluminescence imaging

**Figure 3:**
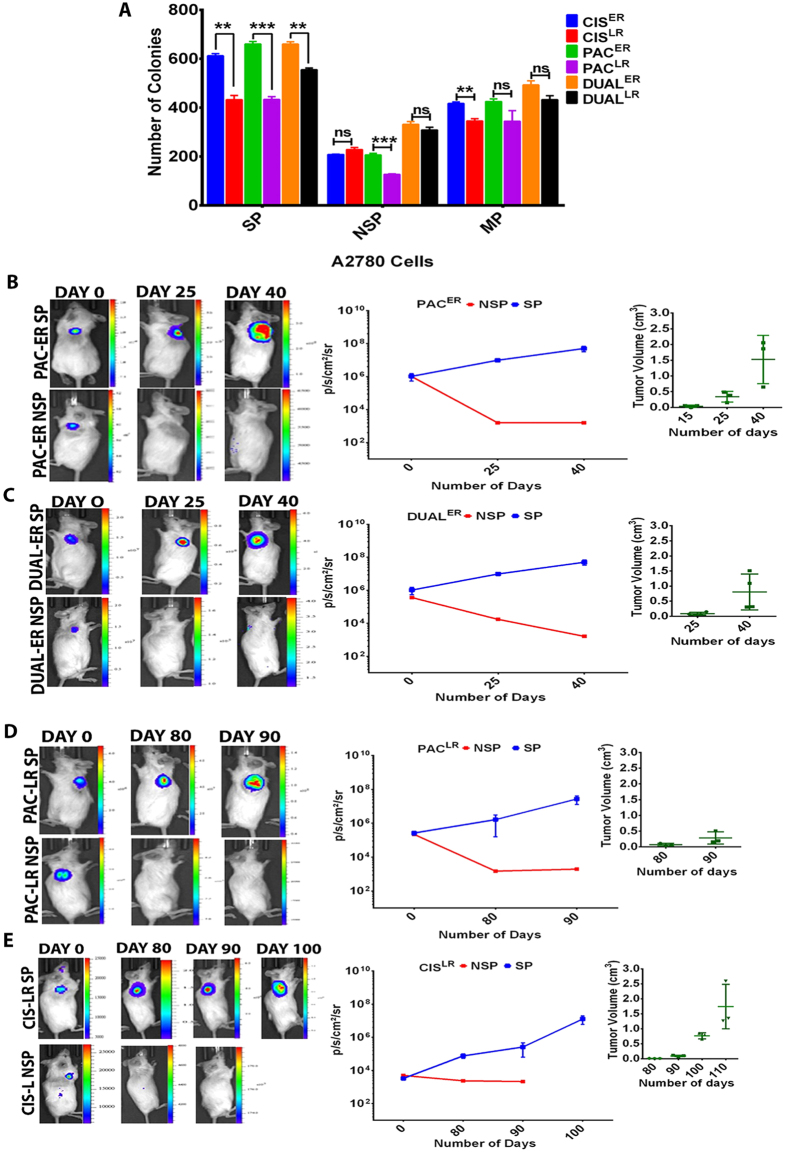
Monitoring tumorigenic properties of SP/NSP cells from early and late resistant stages of A2780 resistant models in real time. (**A**) Bar graph showing significant increase in the clonogenic potential of ER-SP cells than LR-SP cells. (**B**,**C**) Representative bioluminescence images of early resistant cells (Pac^ER^ and Dual^ER^) from day 0 to day 40 and graphical representation showing increased bioluminescence signal and tumor volume by SP cells. NSP cells showed significant decrease in bioluminescence and absence of tumor formation (p < 0.05), [Data represented as ± SEM for n = 3]. (**D**,**E**) Representative bioluminescence images of late resistant cells (Pac^LR^ and CIS^LR^) from day 0 to day 90 and graphical representation showing increased bioluminescence signal and tumor volume by SP cells. NSP cells showed significant decrease in bioluminescence signal and absence of tumor formation (p < 0.05) [Data represented as ± SEM for n = 3].

The most crucial characteristics that differentiate a cancer stem cell from a normal stem cell lies in its inherent tumorigenic ability. After assessing the self-renewal and differentiation properties, we sought to evaluate the tumorigenic potential of the SP population through *in vitro* clonogenic assay and *in vivo* tumor forming ability in immune compromised mice. As expected the NSP cells from both early and late resistant stages of all the three models of A2780 cells formed significantly lower clones than their SP counterparts. Number of clones formed by the NSP cells was also found to be lower than the number of clones formed by total population across all the resistant models (Fig. 3A). Interestingly SP cells from each of the early resistant stages (Cis^ER^, Pac^ER^ and Dual^ER^) showed significantly higher number of colonies than SP cells from late resistant stages (Cis^LR^, Pac^LR^ and Dual^LR^) (Fig. 3A). This intriguing differential clonogenic property exhibited by early and late resistant SP cells was further tested in living subjects in real time.

Fifty thousand SP and NSP cells from Pac^ER^ and Dual^ER^ A2780 cells were sub-cutaneously implanted in both flanks of NOD/SCID mice (n = 5) and longitudinally monitored by bioluminescence imaging. To directly compare the tumorigenic ability of CSC (SP cells) and non-CSC (NSP cells) population of different resistant stages, we purposely implanted higher number of cells to measure the actual tumorigenic potential (even with lower efficiency) of the NSP populations in the same mouse. Though luciferase signal was observed at both SP and NSP sites in all 10 mice at day 0, tumor development was observed in 60% and 80% of mice for Pac and dual resistant cells respectively and only at SP cell implantation sites. Enhanced luciferase signal at SP-Pac^ER^ tumor site (n = 3) was observed from day 0 to day 25 (2.21 × 10^5^ ± 9.24 × 10^4^ to 8.67 × 10^6^ ± 5.80 × 10^6^ p/sec/cm^2^/sr) indicating tumor initiation which increased (3.54 × 10^8^ ± 2.05 × 10^8^ p/sec/cm^2^/sr) and palpable tumors (0.98 ± 0.80 cm^3^) were observed by day40 (Fig. 3B). In contrary, the NSP-Pac^ER^ tumors lost the bioluminescence signal over time and did not show any palpable tumor till 50 days. Similar trends were observed for SP-dual^ER^ and NSP-dual^ER^ tumors, where bioluminescence signal at SP implantation sites increased from day 0 to day 25 to day 40 (1.04 × 10^6^ ± 5.06 × 10^5^ to 9.84 × 10^6^ ± 2.1 × 10^5^ to 5.06 × 10^7^ ± 1.76 × 10^7^ p/sec/cm^2^/sr) with palpable tumors (0.57 ± 0.25 cm^3^) at day 40 were found only for SP cells (Fig. 3C).

Quite distinct and unexpected tumor growth kinetics was observed when SP-Cis^LR^ and SP-Pac^LR^ A2780 tumors (n = 5 each) were monitored in real time. To characterize late resistant SP population independent of nature of drug used, the cisplatin resistant cells were used along with paclitaxel resistant cells. For both SP and NSP tumor sites, no increase in bioluminescence signal was found from day 0 to day 50 (data not shown). Unlike SP-Pac^ER^ cells where tumor initiation noticed from day 25, first detectable luminescence signal from SP-Pac^LR^ tumors was found on day 80 (2.65 × 10^5^ ±  6.37 × 10^4^ to 1.65 × 10^6^ ± 1.49 × 10^6^ p/sec/cm^2^/sr) and a sharp increase in bioluminescence was observed within 10 days (2.73 × 10^7^ ± 1.43 × 10^7^ p/sec/cm^2^/sr) (Fig. 3D). No tumors or bioluminescence was observed at the site of NSP cells implantation. Compared to SP-Pac^ER^ cells, SP-Pac^LR^ cells produced significantly smaller tumors (SP PAC^ER^ = 1.52 ± 0.76 cm^3^; SP PAC^LR^ = 0.28 ± 1.9 cm^3^). For SP-Cis^LR^ tumors, detectable signal was found on day 80 (7.60 × 10^4^ ± 2.26 × 10^4^ p/sec/cm^2^/sr) followed by gradual increase in signal (1.27 × 10^7^ ± 6.7 × 10^6^ p/sec/cm^2^/sr) and tumor volume (0.76 ± 0.058 cm^3^) till day 100 (Fig. 3E). Again loss of bioluminescence signal and no tumor formation were observed at NSP cells implantation sites. Only 60% mice for both these group of late resistant cells developed tumors.

The higher tumorigenic potential found in SP cells of early resistant stages is quite unexpected and might be driven by an active signalling cascade involved in cellular proliferation. We have previously reported that irrespective of the nature of the drugs, the early resistant cells possess enhanced expression of IGF-1R which decreases at late stages of resistance^19^. Thus this crucial cell proliferating signalling cascade governed by IGF-1R might have influence on the enhanced tumorigenic potential of SP cells of early resistant stages.

### Effect of IGF-1R inhibition upon cancer stem cells and chemoresistant phenotype

**Figure 4:**
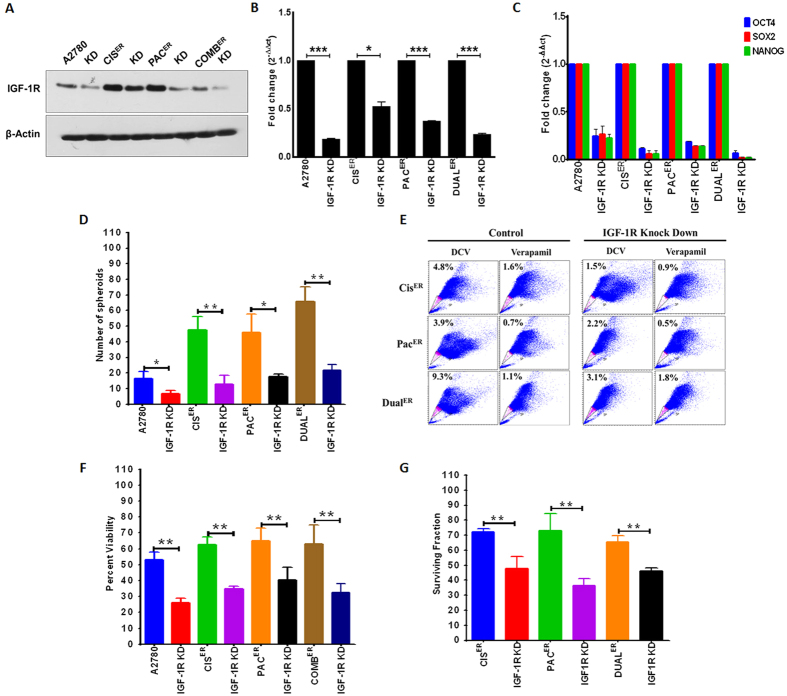
Effect of IGF-1R inhibition upon CSC and resistance phenotypes at early stages of all cellular resistant models. (**A**,**B**) Western blot analysis and RT PCR showing marked decrease in the levels of IGF-1R in A2780-Cis^ER^, A2780-Pac^ER^ and A2780-Dual^ER^ IGF-1R knockdown cells compared to the respective controls. (**C**) Bar Graph showing fold decrease in expression of pluripotent genes (*oct4, sox2* and *nanog*) in A2780-IGF-1R knockdown cells compared to their parental cells where dual^ER^ knockdown cells showed maximum down regulation. (**D**) Graphical representation of spheroid forming ability of A2780, Cis^ER^, Pac^ER^ and Dual^ER^ control cells and their respective IGF-1R knockdown cells. (**E**) MTT assay showing marked decrease in the cell viability post drug treatment (IC_50_) in the A2780-IGF-1R KD cells. (**F**) Bar Graph showing decreased surviving cells in A2780-IGF-1R KD cells post drug treatment (IC_50_) compared to their parental cells. (**G**) FACS dot plot showing decreased SP cells after knockdown of IGF-1R cells compared to their respective control cells.

Till date, association of IGF-1R signaling with CSC phenotype is reported for colon and breast cancers^22^,^23^. Whether and how IGF-1R signaling influences the stemness phenotype in chemoresistant ovarian cancer cells is never investigated. We next attempted to investigate the association of upregulated IGF-1R with CSC features by inhibiting IGF-1R with different strategies and testing the various properties of CSCs. Treatment with Picropodophyllin (PPP), a small molecule inhibitor of IGF-1R decreased spheroid forming ability and pluripotent gene expression in sensitive, ER and LR cells from different resistant models of A2780 cells (Supplementary Figure 2). Maximal inhibition in spheroid formation and stemness gene expression was observed in early resistant stages compared to sensitive and late resistant stages except for the dual resistant model where reduction in levels of spheroid formation did not differ between early and late resistant stages. Decreased stemness properties by PPP in late resistant cells were unexpected and could have caused by overall inhibition of IGF-1R signaling. Additionally, PPP, though specifically inhibits IGF-1R over insulin receptor can exert some toxicity through microtubules inhibition^24^ which prompted us to adapt shRNA mediated stable knockdown strategy for early resistant stages of A2780 cellular models. Western blot analysis and RT PCR showed significant decrease in IGF-1R level in knockdown cells (Fig. 4A,B). Silencing of IGF-1R resulted in drastic decrease in the levels of pluripotent gene expression (*oct4, sox2* and *nanog*) compared to their parental cells and this decrease was maximally observed in Dual^ER^ cells (Fig. 4C). The effect of IGF-1R knockdown was even more prominent on self-renewal ability, showing significant decrease in their spheroid forming capacity (A2780 sensitive cells~2.5 fold, Cis^ER^~3.66 fold, Pac^ER^~2.6 fold and dual^ER^~3.04 fold) and SP phenotype (Cis^ER^~3.2 fold, Pac^ER^~1.7 fold and dual^ER^~3 fold) (Fig. 4D,E). IGF-1R silencing in early resistant cells also affected their chemoresistant phenotype as observed by decrease in percent viability and clonogenic potential (Fig. 4F,G). Since late resistant stages possessed very little IGF-1R expression (Singh *et al*.)^19^ our attempt to silencing did not result in further decrease (data not shown).

### A feedback loop in IGF-1R-AKT axis influence resistance maintenance and CSC features

**Figure 5:**
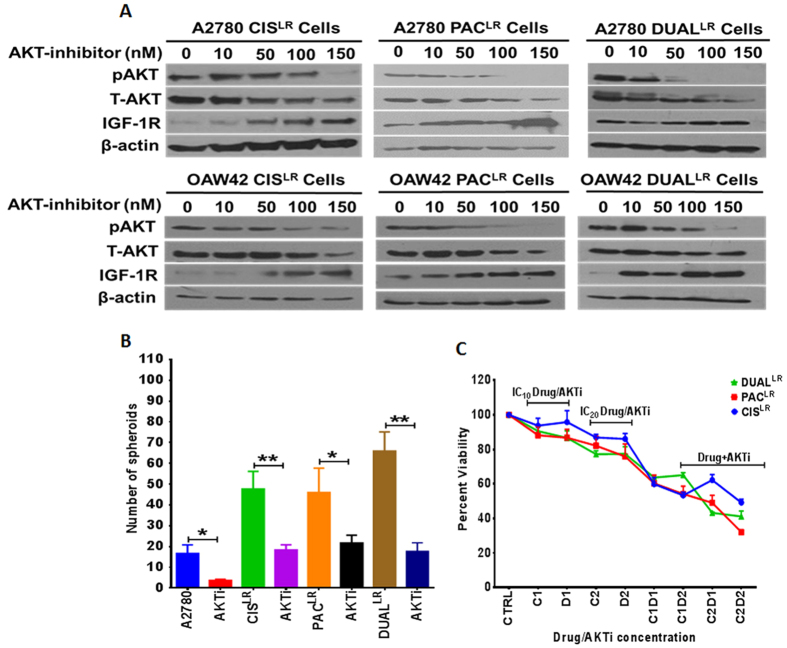
Effect of AKT inhibition upon CSC and resistance phenotypes at late stages of all cellular resistant models. (**A**) Western blot analysis showing AKT inhibitor treatment led to decreased levels of pAKT with increasing IGF-1R levels in a dose dependent manner across all the late resistant cells (A2780 & OAW42). β actin was used as a loading control. (**B**) Decreased spheroid forming ability of late resistant cells across all the resistant models (cisplatin, paclitaxel and dual) after treatment with AKT inhibitor (150 nM). (**C**) MTT assay showing least percent viability for all the late resistant A2780 cells after combinatorial treatment of AKT inhibitor and respective drugs (at IC_20_ concentrations) in comparison to treatments of inhibitor and drug alone (at respective IC_10_, IC_20_ concentrations) or combinatorial treatment at IC_10_ concentrations. (Data represented as ± SEM, *p < 0.05; **p < 0.005).

In spite of low IGF-1R expression, late resistant cells of all the different drug resistant models have high levels of phosphorylated AKT^19^ indicating that AKT could be a critical player in the maintenance of both chemoresistance and CSC phenotype. Indeed treatment with an AKT inhibitor decreased p-AKT levels in a dose dependent manner and also spheroid formation in Cis^LR^, PAC^LR^ and Dual^LR^ A2780 cells (Cis^LR^~2.6 fold, Pac^LR^~2.12 fold and dual^LR^~3.8 fold) (Fig. 5A,B). Levels of total AKT also slightly decreased after treating the cells with high concentration of the inhibitor (150 nm). Intriguingly AKT inhibition resulted in marked increase in IGF-1R levels (Fig. 5A), suggesting of a possible feedback loop in the IGF-1R-AKT axis during development of chemoresistance in ovarian cancer cells. Similar trend was also seen in other drug resistant models (cisplatin, paclitaxel and Dual) of OAW42 cells. Since we observed a feedback loop in IGF-1R-AKT axis, an AKT inhibitor alone and in combinations of drugs (cisplatin, paclitaxel and cisplatin + paclitaxel)at lowest possible doses (IC_10_ and IC_20_ concentrations) was used to treat the cellular models. Maximum cell kill (Cis^LR^ = 50.79%; Pac^LR^ = 68% and Dual^LR^ = 59%) was observed in combinatorial treatment of the inhibitor and IC_20_ concentrations (Cis^LR^ = 2 ug/ml, PAC^LR^ = 50 ng/ml and Dual^LR^ = 7 ng/ml cisplatin + 40 ng/ml paclitaxel) of drug/s in comparison to IC_10_ combinatorial treatments as well as drugs and inhibitor alone (Fig. 5C). Exertion of a negative feedback loop upon an upstream molecule (IGF-1R) by a downstream member (Akt) of the same signalling cascade prompted us to analyse the efficacy of combinatorial treatment of IGF-1R and AKT inhibitors in late resistant cells. Significantly higher cell death (~2 fold) and reduced IGF-1R and pAkt levels were observed in combinatorial treatments (IC_20_ doses of PPP and AKT inhibitor) than single treatments as measured through MTT assay and western blotting (Supplementary Figure 4A,B).

## Discussion

Presence of stem cell like population in various malignancies and their enrichment as the disease turns radio/chemoresistant is a challenging affair for successful treatment. The quiescent and resistant nature of the cancer stem cells act as double edged sword to battle the therapeutic effects of cytotoxic drugs especially for those which target replication machinery of the cells. Understanding and identifying signalling pathways critical for CSC functionality is therefore an active area of current biomedical research. Though acquirement of chemoresistance is a common problem for majority of the cancers, it is particularly devastating for epithelial ovarian cancer patients. Several recent studies suggest the presence and deleterious effect of CSCs in this chemoresistant patient population^9^,^10^. Acquirement of resistance towards drug is a dynamic and multifactorial process and dominated by enrichment of CSC population. To understand this intricate relation between CSCs and enhanced drug resistance, we have used dynamic cellular models of resistance developed indigenously against Cisplatin, Paclitaxel and Dual drugs in A2780 and OAW42 ovarian cancer cells^20^. Based on the resistance indices, these cellular models are categorized into early and late resistant stages and shown to possess preferential up regulation of IGF-1R and activated AKT at early and late resistant stages respectively^19^. We hypothesized that not only the number but the properties of CSCs might enhance with increasing chemoresistance and targeting these population at right stages could be therapeutically beneficial. Thus in this study, we assessed two crucial functional properties (self-renewal and differentiation) of cancer stem cells through side population and spheroid formation assays and compared their *in vivo* tumorigenicity at early and late resistant stages of A2780 chemoresistant models. As expected significant enrichment of SP population was observed across all the resistant models (cisplatin, paclitaxel and Cisplatin + Paclitaxel) in both cell lines. Self-renewal is a central character of both normal and cancer stem cells to maintain their own pool which is assessed by spheroid forming capabilities. Increased spheroid forming ability from early to late resistant cells indicated that self-renewal properties of cancer cells dynamically enhance with increased drug resistance. Co-ordinated protein-protein interaction of Oct4 and Sox2 transcription factors initiates Nanog transcription and these three proteins in coordination are thought to be central regulators of several other genes that balance self-renewal and differentiation. Intriguingly, expression of *oct4* and *sox2* significantly increased from sensitive to early resistant stages and then remained plateau till late stages of resistance suggesting that an early up regulation of pluripotent gene expression is essential for maintenance of both self-renewal and differentiating ability of CSCs. An apparent trend of enhanced spheroid formation in paclitaxel resistant cells compared to cisplatin resistant cells might result from increased pluripotent gene expression at early stage. However, a detail investigation is required to understand the drug specific effects of pluripotent gene expression and self-renewal ability.

Intriguingly, despite of similar levels of IGF-1R expression, the CIS^ER^ cells possess lower expression of all the three pluripotent genes than PAC^ER^ cells. Several signaling pathways (LIF/Stat3, Wnt/GSK3β and TGFβ/Smad3) that regulate pluripotent gene expression in a context dependent manner exhibit cross talk with IGF-1R signalling^25^. It is still not known how and to what extent upregulated IGF-1R influences these pathways in cisplatin and paclitaxel resistance. However, it is well known that response of ovarian cancer cells to platinum and taxol drugs are variable through differential gene signatures^26^. Earlier publication from our lab showed that induction of NF-kβ is essential for maintenance of only cisplatin resistance but not for paclitaxel resistance at late stages^20^. Thus, it is possible that a co-operative effect of IGF-1R along with other regulatory molecules exert differential levels of activation of the pluripotent genes during diverse drug resistance.

Higher spheroid formation and successive differentiation of SP cells to chemosensitive NSP lineages indicated that the SP fractions are enriched with CSC population. Ovarian Cancer Stem Cell biomarkers (CD133 and CD44) also showed increased expression with increasing resistance. However heterogeneity lies within these two cellular resistant models where A2780 and OAW42 showed differential biomarker expression. A2780 resistant model showed increased CD133 expression without a detectable expression of CD44. On the other hand, OAW42 cellular resistant models showed incremental CD44 expression and minimal levels of CD133. When tumorigenic ability of SP population from early and late resistant stages of A2780 cells was assessed in real time by optical imaging, faster tumor formation was observed in early resistant groups. Since these early resistant cells possess elevated IGF-1R expression, inhibition of IGF-1R by a small molecule inhibitor or specific shRNA significantly diminished SP population, spheroid formation as well as pluripotent gene expression. Intriguingly, when the late resistant cells were challenged with an AKT inhibitor, stemness features were declined and IGF-1R expression was elevated. Thus our data suggests that dynamic changes in chemoresistance development in ovarian cancer cells influence functionality of CSC pool which is tightly regulated by IGF-1R-AKT signalling cascade.

Insulin like growth factor 1 receptor signals through PI3K-AKT or MAPK-Erk pathway to directly control the cellular proliferation via activation of a series of protein kinases during the course of developmental process^1^,^27^. This upregulated IGF-1R signaling has also been shown to have a critical role in acquirement of chemoresistance^5^,^6^,^19^ and CSC phenotype primarily in human breast cancer and hepatocellular carcinoma^23^,^28^.

Herein we for the first time elucidated the role of IGF-1R signaling in enrichment of CSC phenotype during acquirement of chemoresistance in ovarian cancer cells. Though acquirement of drug resistance involves many molecular and biochemical changes in cellular machineries, a class of transporter proteins known as multi drug resistance proteins plays key role in diminishing chemotherapeutic effects. Side population assay, a classical method to measure drug efflux properties of cells, has been adapted to assess CSC enrichment in various studies^29^,^30^,^31^,^32^,^33^. The SP cells showed higher cisplatin/paclitaxel/dual drug resistance than the main and NSP cells and higher spheroid formation and pluripotent gene expression in our models. Intriguingly, occurrence of more than 50% of NSP cells in repeatedly sorted and successively cultured SP cells indicated differentiation abilities of these CSCs. Till date, neuronal, haematopoietic and cancer stem cells were characterised for lineage specific differentiation^34^. A recent study by Touil *et al*.^35^ using Rhodamine 123 (Rh123) exclusion assay (similar to SP assay) showed that a small subset of (Rh123)low) cells from metastatic human melanomas and melanoma cell lines is enriched for stem cell like features and can produce non-stem (Rh123(high)) progeny and melanosphere^35^. Our study thus provides a similar evidence of differentiation ability of ovarian cancer stem cells into non-stem and relatively drug sensitive progeny. In addition reduction in IGF-1R expression through small molecule inhibitor or shRNA predominantly decreased the stemness features in early resistant cells, while inhibition of AKT diminished spheroid formation in late resistant cells indicating an intricate influence of IGF-1R-AKTsignalling on cancer stem cell functionalities.

In accordance with cancer cell heterogeneity residing in single tumor, diversity in cancer stem cell population in the same tumor or cell line has been identified^13^,^34^. This heterogeneity is majorly identified through presence or absence of biomarkers in conjugation with ALDH assay^17^. The biggest disadvantage of biomarker based segregation is the inability of utilizing them for targeting due to their presence in normal cells. Active attempts are being made to understand and utilize the targetable molecules in the diverse population of CSCs. High claudin 4 expression in patient derived CD44^+^ ovarian CSCs was shown to be a potential target for *Clostridium perfringens* enterotoxin^18^. The intrigue observation of faster tumorigenicity by SP fraction of early resistant cells (A2780 Pac^ER^ and dual^ER^) cells compared to their late resistant counterparts as well as other late resistant SP cells (A2780 Cis^LR^ and Pac^LR^) in NOD/SCID mice demonstrates existence of functional heterogeneity in CSC population of the same cellular model. This heterogeneity does not depend on the nature of drug since early resistant cells from both Paclitaxel and dual resistant model showed similar rate of tumor formation. Real time monitoring of tumor growth by optical imaging conclusively demonstrated the non-tumorigenic nature of the NSP cells isolated from early as well as late resistant cells of A2780 chemoresistant models. While an upregulated IGF-1R expression could be a plausible factor for faster tumorigenic nature of the early resistant cells, the molecular factors behind the slower tumorigenic potential of late resistant cells are yet to be identified. Predominant presence of activated AKT contrasts this relatively dormant nature of late resistant cells, however, higher spheroid forming ability indicate greater degree of cellular quiescence and slower proliferation in these highly resistant population. This slow proliferative nature of the late resistant A2780 cells was reported earlier by us^20^. Recently, a rare subpopulation melanoma Rh123l^ow^ stem like cells existing in quiescent and slow cycling stage showed to possess higher proportion of activated AKT compared to their Rh123^high^ counterpart cells^35^. Phosphorylated AKT is known to control cellular quiescence through HIF1α and c-Myc inactivation and repression of oxidative phosphorylation^36^,^37^,^38^. It could be possible that the late resistant cells in our models are more quiescent and slow cycling in nature and thus exhibit slower tumor proliferation. Further study to identifying the exact mechanism is in progress. Re-appearance of IGF-1R post AKT inhibition signifies presence of a feedback loop in these resistant models which is independent of nature of drug or cell lines. Previous report of such feedback loop in breast cancer cells showed that AKT inhibition resulted in up regulation of HER2, HER3, IGF-1R and INSR expression and downstream signaling^39^. As expected, treatments with IGF-1R and Akt inhibitors led to reduced cell growth and IGF-1R expression possibly due to interruption in the feedback loop in IGF-1R-Akt signalling. We speculate that such dual inhibition for members of same signalling cascade exhibiting feedback loop could be a more effective therapeutic strategy. Interestingly, combinatorial treatments of AKT inhibitor and chemotherapeutic drugs at low concentration (IC_10_ & IC_20_) also showed significant decrease in viability in late resistant cells possibly due to an overall inhibition of IGF-1R-AKT signaling.

Recent evidences supporting the link between CSC and therapy resistance open the possibility of targeting resistant population as an approach to CSC eradication. Our study demonstrates existence of IGF-1R-AKT signaling mediated functional heterogeneity in the ovarian CSC population which causes differential tumorigenic ability in living subjects. Irrespective of the nature of drugs, this IGF-1R-AKT axis bestows a feedback loop during generation of chemoresistance. Our report thus specifies IGF-1R-AKT signaling as a prime determinant of cancer stem cell functionality and chemoresistance and a potential therapeutic target axis in ovarian carcinoma.

## Materials and Methods

### Cell Culture

A2780, IGF-1R knockdown A2780 and OAW42 cells were cultured in DMEM and MEM medium respectively supplemented with 10% foetal bovine serum and 1% penicillin-streptomycin.

### Spheroid formation

Spheroids were generated in low adherent 24-well dishes using 2000cells/well in special medium (serum devoid DMEM or MEM complemented with FGF (20 ng/ml), EGF (10 ng/ml), Insulin (20 ng/ml), LIF (10 ng/ml) and 0.1% pen-strep) and cultured till 3^rd^ passage. Quantitative assessment of spheroids was blindly performed by counting them under microscope. For the successive sphere-forming assay, cells from primary spheres were collected by centrifugation, dissociated with trypsin-EDTA and mechanically disrupted with a pipette. Two thousand single cells were proceeded for sphere-forming assay.

### Western blotting

Western blotting was performed as described earlier^19^. Antibodies against IGF-1R β-subunit, AKT, pAKT and beta actin were from Cell Signaling Technology.

### Quantitative real-time PCR

Two micrograms of total RNA was reverse transcribed with cDNA synthesis kit (Invitrogen). RT PCR was performed using SYBR Green method (Invitrogen) and appropriate gene specific primers and GAPDH as normalization control.

### FACS

Side and Non Side population cells were sorted using Dye Cycle Violet (DCV) dye (Invitrogen)^40^ and BD FACS Aria tagged with violet laser. Membrane drug transporter blocker Verapamil (50 μM; Sigma) was used as negative control and gating. Data analysis was performed through DIVA software. Cell surface biomarker (CD44 and CD133) analysis was performed with FlowJo version 10 software. Anti CD44 and Anti CD133 antibodies were procured from Cell Signaling Technologies and Abcam respectively.

### Lentivirus production

Lentiviruses carrying IGF-1R target sequence (5′AGACCTGAAAGGAAGCGGAGA-3′)^41^ were produced in 293FT cells by transfection with lentivector plasmid, P-delta packaging plasmid, VSVG envelope protein plasmid (4:2:1 ratio) and lipofectamine (Invitrogen). Viruses were collected post 60 hours of transfection. Early cisplatin, paclitaxel and dual drug resistant cells were transduced with lentiviruses and stable cells expressing shIGF-1R constructs were sorted using GFP.

### MTT cell cytotoxicity assay

To evaluate cytotoxicity of various chemotherapeutic drugs (cisplatin, paclitaxel and combination), 2 × 10^3^ cells were seeded in 96 well plates (Corning, USA). Cells were exposed to different concentrations of drugs for 48 hours. Cell viability was assessed using the MTT (3-[4,5-dimethylthiazol-2-yl]c-2,5-diphenyltetrazolium bromide) (Sigma, USA).

### Bioluminescence imaging

All of the experiments were approved by the Institutional animal ethics committee of ACTREC and were performed in accordance with the approved guidelines. Fifty thousand SP & NSP cells were injected subcutaneously on two shoulders of 6–8 weeks old NOD/SCID mice. Bioluminescence imaging was performed by injecting D-Luciferin substrate (30 mg/ml) on the day 0 and subsequently to monitor tumor initiation and progression using IVIS Spectrum (Perkin Elmer). The mice were maintained under isoflurane (Foreknew^®^, ChoongWae Co., Korea) anaesthesia during the entire process. Data analysis was performed using Living Image 4.4 software. Tumor volume was measured using Vernier calliper and the calculated by the formula (Tumor volume = ½ x Length x (Width)^2^.

### Statistical Analysis

Data represent the mean ± SEM of at least three independent experiments and were analysed as a biological replicates for significance using unpaired Student’s t test. P value ≤ 0.05 was considered as significant.

## Additional Information

**How to cite this article**: Singh, R. K. *et al*. An active IGF-1R-AKT signaling imparts functional heterogeneity in ovarian CSC population. *Sci. Rep.* **6**, 36612; doi: 10.1038/srep36612 (2016).

**Publisher’s note:** Springer Nature remains neutral with regard to jurisdictional claims in published maps and institutional affiliations.

## Supplementary Material

Supplementary Information
