# Supplementary material for: An active IGF-1R-AKT signaling imparts functional heterogeneity in ovarian CSC population
Source: Sci Rep. 2016 Nov 7;6:36612. doi: 10.1038/srep36612 (PMC5098199; doi:10.1038/srep36612)

Supplementary Information

Title: An active IGF-1R-AKT signaling imparts functional heterogeneity in ovarian CSC population

Ram K Singh[1], Ajit Dhadve[1], Asmita Sakpal[1], Abhijit De[2] and Pritha Ray[1]*

[1]Imaging Cell Signaling and Therapeutics Lab, Advanced Centre for Treatment, Research and Education in Cancer (ACTREC), Tata Memorial Centre, Navi Mumbai, Maharashtra, India

[2]Molecular Functional Imaging Laboratory, Advanced Centre for Treatment, Research and Education in Cancer (ACTREC), Tata Memorial Centre, Navi Mumbai, Maharashtra, India

* To whom request for reprints should be addressed

E-mail: pray@actrec.gov.in

Supplementary material

Supplementary Table 1: List of primer sequences.

IGF-1R (FORWARD)	CTGGACTCAGTACGCCGTTT
IGF-1R (REVERSE)	GGAACTGAAGCATTGGTGCG
OCT4 (FORWARD)	GTGGAGAGCAACTCCGATG
OCT4 (REVERSE)	TGCAGAGCTTTGATGTCCTG
SOX2 (FORWARD)	AACCCCAAGATGCACAACTC
SOX2 (REVERSE)	GCTTAGCCTCGTCGATGAAC
NANOG (FORWARD)	AAAGCTTGCCTTGCTTTGAA
NANOG (REVERSE)	AAGTGGGTTGTTTGCCTTTG
GAPDH (FORWARD)	TGCACCACCAACTGCTTAGC
GAPDH (REVERSE)	GGCATGGACTGTGGTCATGAG

Supplementary Table 2: Percentage of SP fraction in A2780 and OAW42 resistant models

	**DCV**	**Verapamil**		**DCV**	**Verapamil**
**A2780**	**1.5%**	**0.1 %**	**OAW 42**	**1.8 %**	**0.1 %**
**CIS^{ER}**	**3.9%**	**0.3 %**	**CIS^{ER}**	**2.0 %**	**0.5 %**
**CIS^{LR}**	**7.2%**	**0.1 %**	**CIS^{LR}**	**4.0 %**	**1.1 %**
**PAC^{ER}**	**5.5%**	**0.3 %**	**PAC^{ER}**	**3.2 %**	**0.8 %**
**PAC^{LR}**	**8.3%**	**0.8 %**	**PAC^{LR}**	**7.4 %**	**0.2 %**
**$DUAL^{ER}$**	**5.05%**	**1.9 %**	**$DUAL^{ER}$**	**4.6 %**	**0.3 %**
**$DUAL^{LR}$**	**19.1%**	**0.7 %**	**$DUAL^{LR}$**	**9.8 %**	**0.1 %**

Supplementary Figure 1: Expression of CSC biomarkers: Expression analysis of CD44 and CD133 across A2780 and OAW42 resistant model showing a gradual increase with increasing resistance.

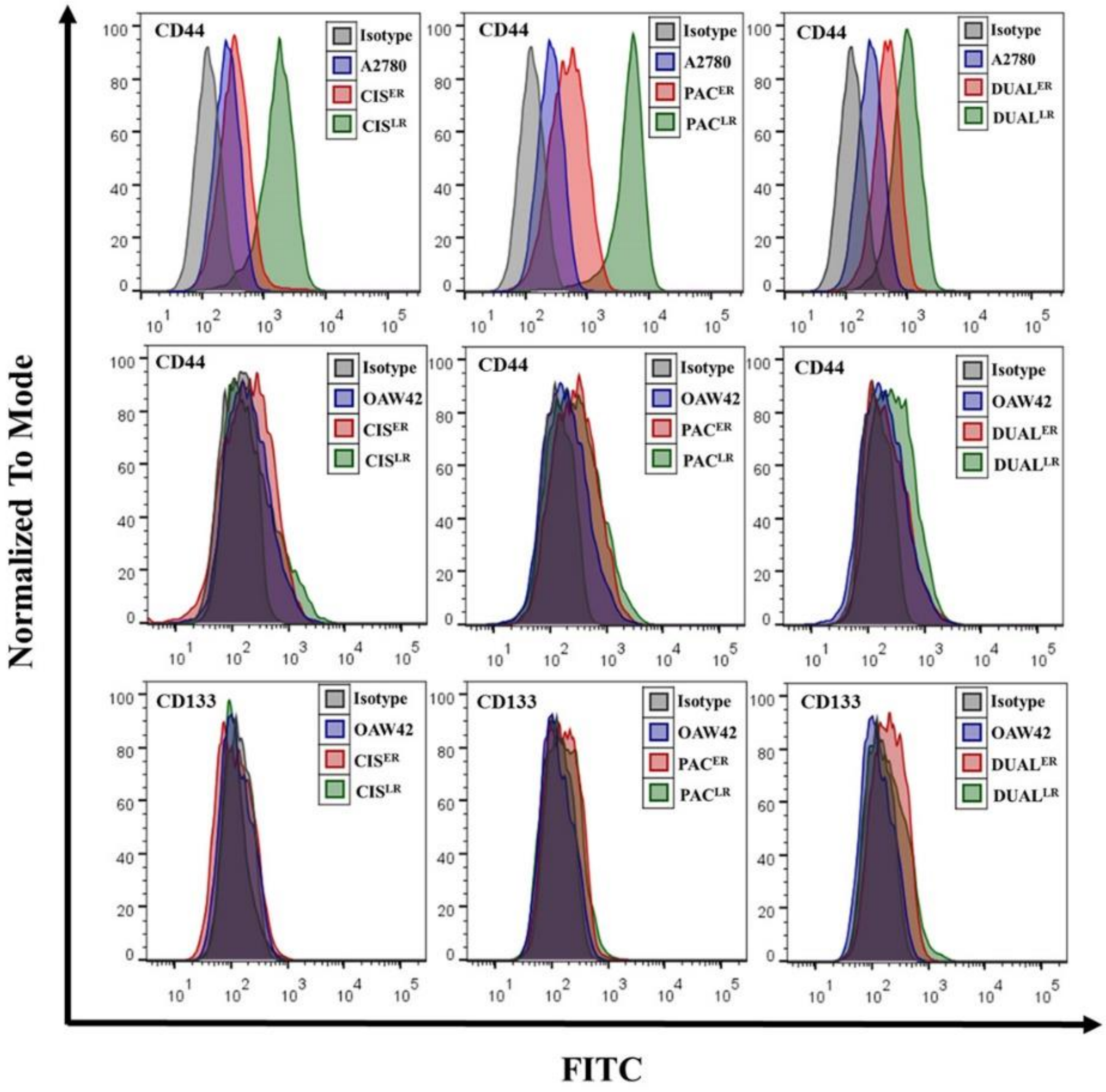

Supplementary Figure 2: Effect of IGF-1R inhibitor (PPP) on CSC phenotype in A2780 resistant models: (A) Expression analysis of pluripotent genes (*oct4, sox2* and *nanog*) after PPP treatment showed significant decrease in all the early and late resistant cells than their respective controls. (B) A similar trend in spheroid forming ability was graphically represented. (C) Table showing fold decrease in spheroid forming property in PPP treated cells.

A

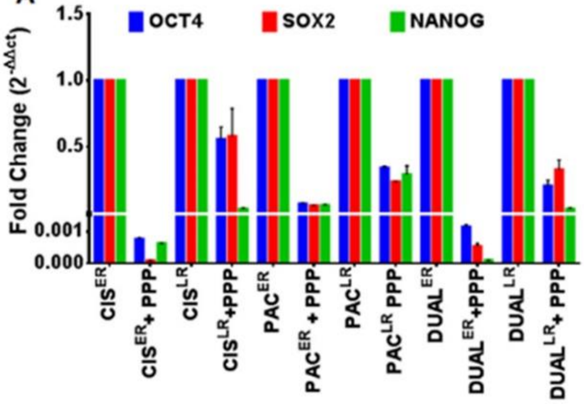

B

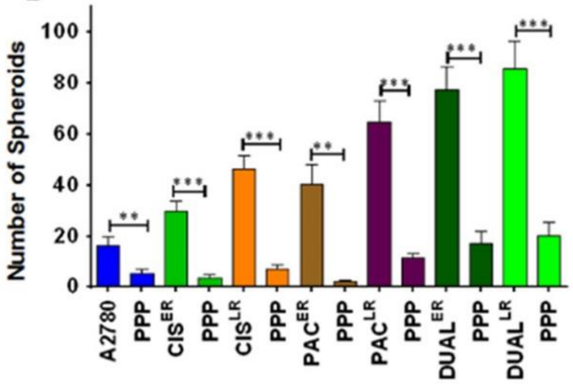

C

Cells	Fold Decrease
A2780	2.8
CIS^{ER}	8.19
CIS^{LR}	6.3
PAC^{ER}	17.3
PAC^{LR}	5.7
$DUAL^{ER}$	4.46
$DUAL^{LR}$	4.2

Supplementary Figure 3: Cell viability assay for SP, MP and NSP fractions during subsequent sorting of SP cells. MTT assay showing significantly higher percent viability by SP cells compared to their respective MP and NSP cells during subsequent sortings (Sort1: SP Vs. NSP; $p<0.0001$, SP Vs. MP; $p<0.001$} {Sort2: SP Vs. NSP; $p<0.0001$, SP Vs. MP; $p<0.001$} {Sort3: SP Vs. NSP; $p<0.001$, SP Vs. MP; $p<0.001$).

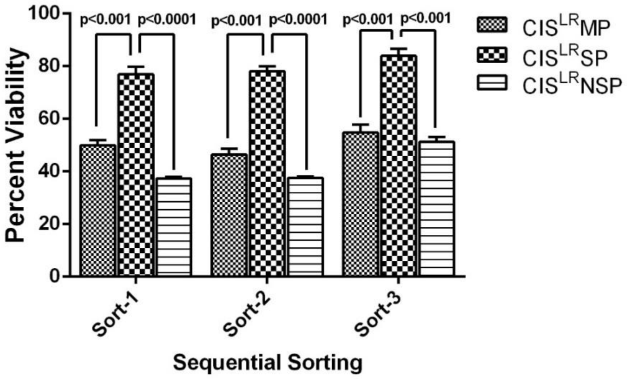

Supplementary Figure 4: Effect of IGF-1R inhibitor (PPP) in combination with AKT inhibitor in late resistant cells of A2780 resistant models:(A) MTT assay showing significant decrease in the percent viability upon combination treatment across the late resistant cells (Cis^{LR}, Pac^{LR} and $Dual^{LR}$). (B) Western Blot analysis showing decrease in the levels of both IGF-1R and pAKT upon combination treatment.

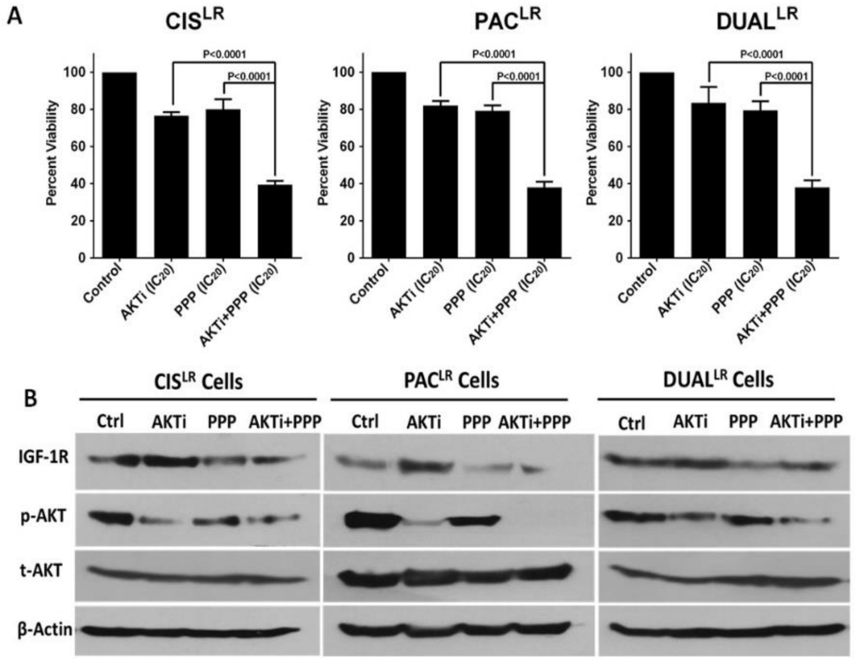

Supplement: Supplementary Information [file srep36612-s1.pdf]
